# The Role of Serum Calprotectin in Defining Disease Outcomes in Non-Systemic Juvenile Idiopathic Arthritis: A Pilot Study

**DOI:** 10.3390/ijms24021671

**Published:** 2023-01-14

**Authors:** Debora Mariarita d’Angelo, Marina Attanasi, Giulia Di Donato, Giuseppe Lapergola, Mariarosaria Flacco, Francesco Chiarelli, Emma Altobelli, Luciana Breda

**Affiliations:** 1 Department of Pediatrics, University of Chieti-Pescara, 66100 Chieti, Italy; 2 Department of Medicine and Aging Sciences, University of Chieti-Pescara, 66100 Chieti, Italy; 3 Department of Life, Public Health and Environmental Sciences, University of L’Aquila, 67100 L’Aquila, Italy

**Keywords:** juvenile idiopathic arthritis, children, serum calprotectin, disease activity, S100 protein

## Abstract

Serum calprotectin (MRP8/14) is currently being studied as a promising biomarker of disease activity and outcome in patients with juvenile idiopathic arthritis (JIA) but the data in the literature are conflicting. The aim of our study was to investigate the potential role of serum calprotectin as biomarker of disease activity and flare/remission in a group of nsJIA patients during a follow-up period of 18 months. In this prospective longitudinal study, two groups of patients with ns-JIA (55 active patients and 56 patients in remission according to Wallace’s criteria) and a control group (50 children) were recruited at baseline from January 2020 to September 2021. JIA patients were followed for up to 18 months at four timepoints: 3 months (T1), 6 months (T2), 12 months (T3) and 18 months (T4). At each timepoint, the following were recorded: JADAS27, blood counts, ESR, CRP, albumin, ferritin and serum calprotectin. To illustrate the performance of calprotectin, Kaplan–Meier curves were constructed from baseline to relapse/remission, dichotomizing patients at baseline in positive/negative on the basis progressive calprotectin cut-offs. Associations between baseline factors and relapse were determined using Cox regression models. Multivariate models were constructed to analyze the effect of covariates. Comparing baseline clinical and laboratory data of the three groups (active vs. inactive JIA vs. controls), only serum calprotectin reached statistical significance (active patients vs. inactive (*p* = 0.0016) and vs. controls (*p* = 0.0012)). In the inactive group, during the 18 months of follow up, 31 patients (55.3%) had a relapse. Comparing the baseline data of relapsers vs. non-relapsers, serum calprotectin showed higher levels (*p* = 0.001) in relapsers. In survival analysis, a log rank test showed significant differences of up to 12 ng/mL (*p* = 0.045). Multivariate Cox regression confirmed that only baseline calprotectin levels were independently associated with disease recurrence. In the active group, in the 12 months of follow-up, 19 patients (38%) entered remission of the disease. In addition, in this group, the only statistical difference at the baseline was the value of MPR8/14 (*p* = 0.0001). Log rank test showed significant differences up to 10 ng/mL (*p* = 0.003). In the multivariate Cox regression, serum calprotectin levels at baseline were independently associated with remission. In conclusion, our study would suggest a dual role for calprotectin in predicting future relapse and treatment response in patients with nsJIA, thus influencing therapeutic decisions and management of these patients during follow up.

## 1. Introduction

JIA is a heterogeneous group of disorders characterized by childhood onset of chronic arthritis of unknown cause [1,2]. In the last two decades, the natural history and prognosis of JIA patients have dramatically improved due to the widespread use of intra-articular corticosteroids, the tendency toward earlier introduction of methotrexate (MTX) and, more recently, the availability of biological medications [1,2,3]. Currently, thanks to the early use of targeted therapies, up to 80% of JIA patients achieve inactive disease in the first year and about 46–57% of non systemic JIA (ns-JIA) patients reach remission within 5 years [4]. However, a high frequency of early flares (50%) after drug discontinuation has been reported in the literature [5,6]. The current method to assess clinical remission in JIA is based on the so-called Wallace criteria, composed of clinical and laboratory variables [7]. However, this method does not directly measure inflammation at primary sites of pathology and may be the subject of confounding influences [8,9]. Indeed, clinical and laboratory markers in use, such as C reactive protein (CRP) or erythrocyte sedimentation rate (ESR), cannot solely detect residual inflammation or therapy response, as several studies have reported [10]. Moreover, both ESR and CRP are acute-phase reactants, which are affected by external systemic factors, requiring second-pass effects (e.g., hepatic production for CRP) and might not directly measure inflammation at the primary site of pathology [10].

Therefore, the identification of new biomarkers or parameters which correlate with disease activity, even subclinical, is an important goal for JIA patients. Current efforts in pediatric rheumatology are aimed to identify biomarkers which, combined with clinical features, might further aid in predicting disease phenotype, severity and course and help refine patient selection for a more targeted treatment [9,10]. Among these interesting biomarker candidates are the members of the calcium-binding S100 leucocyte proteins family, S100A8/9 and S100A12. S100A8/9 or calprotectin (also referred as MRP8/MRP14, calgranulin A/B, L1 protein and cystic fibrosis antigen) is an important proinflammatory factor of innate immunity, which is released during cell activation and turnover [11,12,13], showing a broad spectrum of intracellular and extracellular immunomodulatory properties. The S100A8/S100A9 complex, secreted extracellularly, activates the Toll-like receptor 4 (TLR4) and the receptor for advanced glycation end products (RAGE) pathways. Furthermore, assuming variable molecular configurations, it can interact in a tissue specific manner [11,12,13,14]. In the inflamed synovium, calprotectin is released directly in situ by innate immune activated cells and thus correlates strongly with local disease processes in joints, even when no systemic involvement occurs. Being a small and soluble molecule (24 kDa), it passes quickly into the bloodstream (as measured in plasma and serum), and appears to be an excellent potential biomarker of inflammation, even when subclinical [12,13,14,15]. The role of serum calprotectin has largely been evaluated in adult rheumatoid arthritis (RA). On the contrary, studies on its usefulness as a predictor of disease activity, treatment response and flare in children with JIA are conflicting. Some studies have demonstrated that patients with higher calprotectin levels at the beginning of methotrexate and anti-TNF treatment were more likely to have a better response after 6 or 12 months [16,17,18]. In contrast, a recent Dutch study involving 137 patients with early non-systemic JIA, mostly the RF negative polyarticular subtype, did not confirm these results [19]. In addition, some authors have postulated that the increased calprotectin levels in patients with inactive disease was correlated with subsequent relapse, assuming that this parameter may be used to identify subclinical disease activity [20,21,22,23,24].

To date, few datasets are available on the role of serum calprotectin in JIA, most are derived from studies with small samples and a follow-up of no longer than 12 months. The aim of this study was to investigate the potential role of serum calprotectin as a potential biomarker of disease activity and flare/remission during the follow-up of 18 months in a group of nsJIA patients.

## 2. Results

### 2.1. Study Design

**Figure 1:**
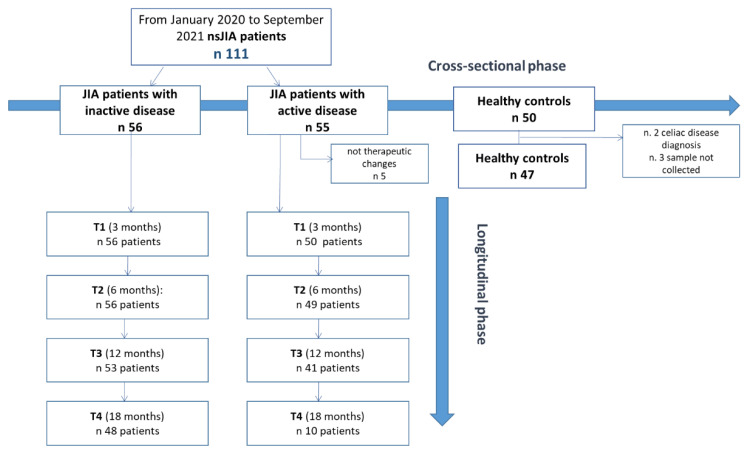
Study design. nsJIA = non-systemic juvenile idiopathic arthritis.

For the study design, refer to Figure 1. Within the inactive JIA patient group, we recorded a lack in follow-up of 14.3% in the 18-month period. In clinically active patients at baseline, five were excluded from the longitudinal phase because they had not introduced any therapeutic intervention and/or therapeutic changes. Since only 10 patients completed the 18-month follow-up (T4), we did not include this timepoint in the statistical analysis. There was a loss in 12-month follow-up of 18% (Figure 1).

### 2.2. Patient Characteristics at Baseline

At T0, the whole “case” study group included 111 JIA children with a higher prevalence of girls (*n* = 92.8, 2.8%). The mean age of the study population was 11.8 ± 4.7 years. A total of 85 patients (76.5%) and 26 patients (23.5%) were *on* therapy and *off* therapy, respectively, at the time of enrollment. Controls included 47 children, with a mean age of 8.77 ± 4.7 years and a lower proportion of girls (*n* = 31, 67.3%).

Table 1 shows the demographic, clinical and laboratory data concerning active and inactive patients and controls. There were no relevant differences in anthropometric and demographic variables between the three groups. We found a statistically significant difference in infiltration history in disease course (*p* = 0.013) and the duration of the biological therapy (*p* = 0.002) between JIA inactive and active patients. Interestingly, we found a significant difference between active and inactive patients in the history of involvement of the TMj (*p* < 0.001). As obviously expected, the JADAS median values differed statistically significantly in the two groups. Although the distribution of JIA subtypes was not equal between the two groups, there was no difference in the treatment regimens.

### 2.3. Laboratory Characteristics and Calprotectin at Baseline

**Figure 2:**
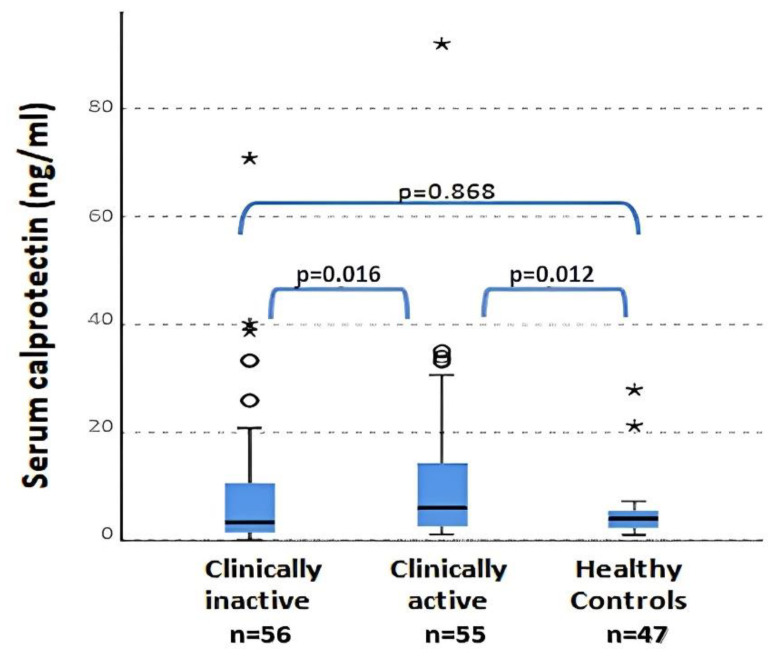
Serum calprotectin levels in active and inactive patients and in controls (ANOVA test). ° = outliers; * = extreme outliers.

No significant differences were found in WBC (*p* = 0.360), ESR (*p* = 0.050) OR ferritin (*p* = 0.610) levels between patients with clinically active and inactive disease and controls. The only parameter that reached significance in the comparison between the three groups was serum calprotectin. Serum calprotectin values were statistically different between clinically active and inactive patients (*p* = 0.016) and between clinically active patients and controls (*p* = 0.012) (Figure 2). No differences between plasma calprotectin levels in the clinically inactive disease group and healthy controls was found (*p* = 0.868). The previous data regarding calprotectin between active and non-active JIA patients were also confirmed by the presence of a positive and significant correlation between the values of calprotectin and JADAS27 (Spearman r = 0.825; *p* < 0.001). Instead, neither CPR (R = 0.077; *p* = 0.456) nor ERS (R = 0.045; *p* = 0.646) presented a correlation with JADAS27. Otherwise, no correlations between serum calprotectin and other flogosis indexes (ESR, CRP, WBC and PLT) were observed in these patients. 

### 2.4. Survival Analysis and Cox Regression Model

We performed the longitudinal phase analyses separately in the two groups of JIA patients, evaluating two different outcomes in the follow-up: the development of a flare in inactive patients at baseline and the achievement of remission after initiation of therapy in clinically active patients at baseline.

In the JIA inactive group, all 56 patients were evaluated at T1 and T2, while 53 were evaluated at T3 and 48 at T4. Among the analyzed patients, 31 patients (55.3%) experienced relapse during the 18-month follow-up: eight patients (14.2%) relapsed at T1, eleven (19.6%) at T2, five at T3 (8.9%) and seven at T4 (12.5%). 

**Figure 3:**
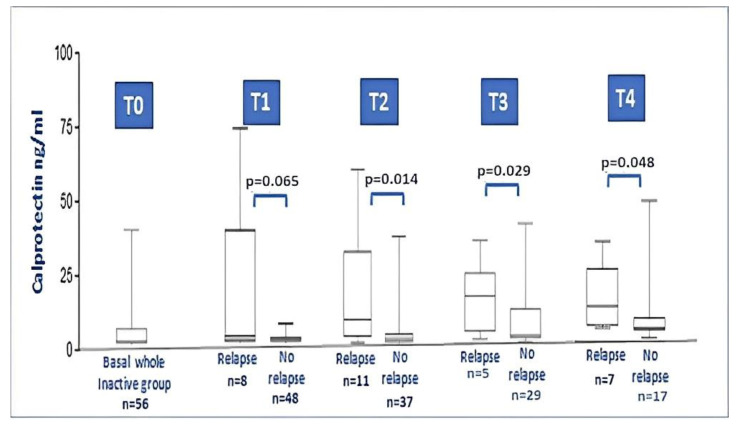
Calprotectin levels in the whole JIA inactive group at baseline and after 3, 6, 12 and 18 months follow-up (relapsers vs. non relapse at each timepoint). At timepoint 2, 3 and 4, the difference between levels of calprotectin in relapser vs. non-relapsers was significant (Mann–Whitney test).

During the 18-month follow-up, comparing the baseline data of JIA relapsers with non-relapsers, no differences between anthropometric and laboratory parameters were found. On the contrary, baseline serum calprotectin showed higher levels in relapsers compared with non-relapsers (8.53 ± 13.7 vs. 1.52 ± 1.58; *p =* 0.001). Figure 3 illustrates the calprotectin level at each timepoint during relapse. No particular difference was found for the type of therapy taken at the time of recruitment, if we either considered the dichotomous variable on/off therapy (*p* = 0.736) or if we considered the distribution of the specific therapy performed (MTX, anti-TNF drugs, no therapy; *p* = 0.546). 

In the JIA active group, all 50 JIA patients reached the first evaluation time point, 49 reached the 6-month follow-up (T2), 41 patients were evaluated at the 12 months follow-up (T3), while only 10 patients reached the last follow-up timepoint. We did not include the latter patients in the statistical analysis given the small size of the sample. Among the patients analyzed, out of the 19 patients (38%) who went into disease remission during the 12 months of follow-up, there were no patients at T1, nine patients (18%) at T2 and five patients at T3 (20%). At the time of recruitment, eight active patients were DMARD naive, while two received more than one biologic drug during the disease course. The recruited patients presented an active disease state for the following reasons: five patients were at JIA onset, thirteen patients were enrolled at a time of arthritis flare after previous remission (mean time of remission was 0.9 ± 2.7 years), three children had a flare-up of arthritis plus uveitis and twenty patients were already previously acute and had never experienced a remission previously. 

During the 12-month follow-up, comparing baseline data of the JIA patients who had gone into remission with those who remained with active disease, no differences between anthropometric, therapeutic and laboratory parameters were found. Serum calprotectin had higher levels in patients in remission than non-remission patients (*p* = 0.001). No difference was found in the type of therapy introduced at baseline (MTX or anti-TNF; *p* = 0.977).

In inactive JIA patients, Kaplan–Meier curves were constructed from baseline to disease relapse to illustrate the predictive performance of calprotectin. Serum calprotectin was dichotomized into high versus low (or positive/negative) according to progressive cut-off levels for optimal prediction of relapse. According to different thresholds for baseline serum calprotectin (3, 10, 12, 15 and 20 ng/dL), the number of patients with high values (or positive) were 27 (48.2%), 13 (23.2%), 10 (17.9%), 9 (16.1%) and 6 (10.7%).

**Figure 4:**
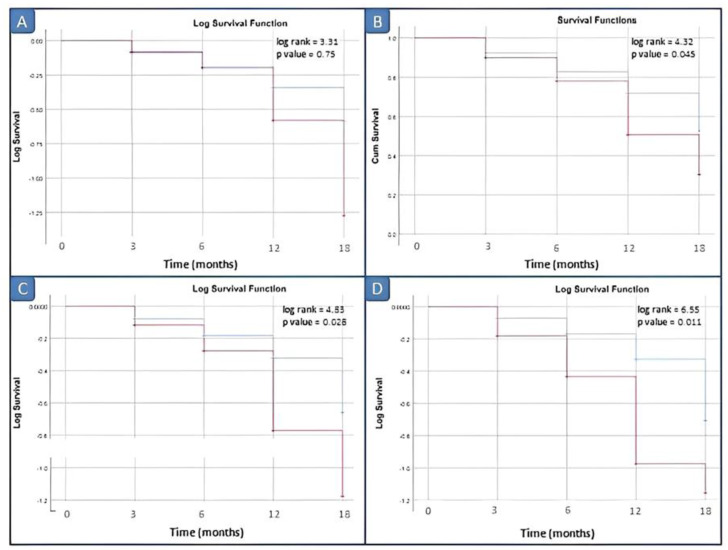
Survival analysis in inactive JIA patients by different cut-off values of calprotectin. (**A**) Cut-off of 10 ng/mL; (**B**) cut-off of 12 ng/mL; (**C**) cut-off of 15 ng/mL; and (**D**) cut-off of 20 ng/mL. Red line: positive patients, i.e., basal calprotectin value ≥ cut off; blue line: negative patients, i.e., basal calprotectin value <cut off.

Kaplan–Meier survival analysis comparing the disease relapse within 3, 6, 12 and 18 months did not demonstrate significant differences between patients with elevated vs. normal serum calprotectin levels for our laboratory kit cut-off value of 3 ng/mL. Survival analyses by log rank tests showed significant differences between patients with elevated vs. normal serum calprotectin levels groups starting from the serum calprotectin value of 12 ng/mL (*p* = 0.045) (Figure 4).

Similarly, in active JIA patients, Kaplan–Meier curves were constructed from baseline to disease remission to illustrate the predictive performance of calprotectin. According to different thresholds for baseline serum calprotectin (3, 10, 12, 15 and 20 ng/dL), the number of positive patients with high values (or positive) were 34 (59.6%), 19 (33.3%), 17 (29.8%), 12 (21.1%) and 11 (19.3%).

**Figure 5:**
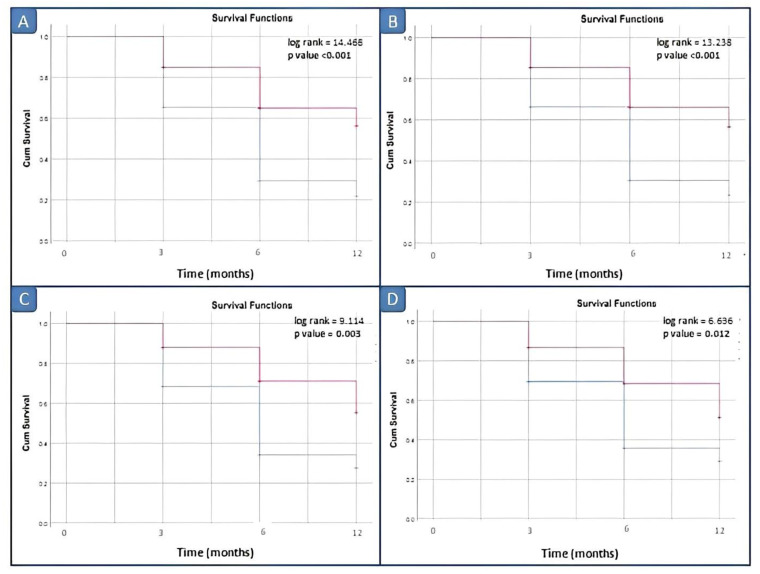
Survival analysis in active JIA patients by different cut-off values of calprotectin. (**A**) Cut-off of 10 ng/mL; (**B**) cut-off of 12 ng/mL; (**C**) cut-off of 15 ng/mL; (**D**) cut-off of 20 ng/mL. Blue line: negative patients, i.e., basal calprotectin value < cut off; red line: positive patients, i.e., basal calprotectin value ≥ cut off.

Kaplan–Meier survival analysis comparing the disease remission within 3, 6 and 12 months did not demonstrate significant differences between patients with elevated vs. normal serum calprotectin levels for our laboratory kit cut-off value of 3 ng/mL. Survival analyses by log rank tests showed significant differences between patients with elevated vs. normal serum calprotectin levels groups starting from the serum calprotectin value of 10 ng/mL (Figure 5).

In inactive JIA patients, univariate Cox regression analysis showed that gender, BMI z score, age at the onset of the disease, disease duration, MTX and biologic drug duration were not associated with disease relapse. On the contrary, the only clinical variable associated with disease relapse was serum calprotectin (at values of 15 and 20 ng/mL, HR 2.02; CI 95% 1.11–3.66 and HR 1.75; CI 95% 1.01–3.02, respectively). In the multivariate regression, only baseline calprotectin levels (3, 10, 12, 15 and 20 ng/dL) were independently associated with disease relapse ((2.09; CI% 1.15–3.81) (HR 1.84; CI 1.05–3.22) (HR 1.91; CI 95% 1.07–3.41) (HR 1.88; CI 95% 1.06–3.33) (HR 2.06; CI 95% 1.07–3.98), respectively).

In active JIA patients, univariate Cox regression analysis showed that gender, BMI z score, age at the onset of the disease, disease duration, MTX duration and biologic drug duration were not associated with disease relapse. On the contrary, the only clinical variable associated with disease relapse was serum calprotectin (at values of 10, 12 and 15 ng/mL: HR 0.44, CI 95% 0.26–0.75; HR 0.44, CI 95% 0.25–0.76; HR 0.44, CI 0.23–0.86). In the multivariate regression, only baseline calprotectin levels (10, 12 and 15 ng/dL) were independently associated with disease relapse ((HR 0.45; CI 95% 0.26–0.78) (HR 0.45; CI 95% 0.25–0.81) (HR 0.43; CI 95% 0.21–0.91), respectively).

The multivariate Cox regression model for inactive and active patients is shown in Table 2.

## 3. Discussion 

To the best of our knowledge, this is the first study with the longest follow-up time evaluating the role of serum calprotectin in both active and inactive JIA patients.

In this prospective longitudinal study, the first finding was that serum calprotectin levels, at baseline, were significantly higher in active patients than in controls and inactive patients, are were further increased with disease activity. In addition, we found that serum calprotectin values correlated strongly with JADAS27 and did not correlate with CRP, ESR, ANA and RF, nor with gender, age or disease duration of patients with JIA, partially in contrast with some previous reports [25,26,27]. In contrast with conventional markers of inflammation, which reflect the acute phase response mediated by hepatocytes or B lymphocytes during inflammation, calprotectin seems to provide additional information on the state of disease in JIA children. These results are in line with previous studies [18,26]. A meta-analysis and systemic review was recently published including 10 specific studies on the pediatric population. Altobelli et al. [27] confirmed that the use of serum calprotectin represents a useful tool in JIA in order to stratify disease activity in JIA children; however, the extreme heterogeneity of the JIA populations studied, especially in terms of treatment, did not allow further conclusive data. In addition, Hurnakova et al. [28] showed that in RA patients, serum calprotectin was the best predictor of ultrasound synovitis, performing better than CRP. In contrast, Romano et al. [29] did not confirm these findings; however, fewer patients were analyzed.

A second major take-away from this study is the utility of serum calprotectin as a potential predictive marker of relapse when measured in patients in remission, as outlined by other studies [20,21,23,30,31,32]. In our cohort study, we showed that during the follow-up, inactive patients, according to different calprotectin cut-off levels, presented different outcomes. We noticed that, as the cut-off increased at baseline, the risk of relapse also increased and the patients with positive values (namely those patients with values above the cut-off threshold considered) showed a higher risk of disease flare-up. In particular, the survival analysis showed that the calprotectin worked particularly well for the value at baseline above 12 ng/mL to detect the next flare during the follow-up. These values seem to be lower than those defined in a similar statistical methodology in RA or JIA [18,23,33,34]. Recently, Inciarte-Mundo et al. [33] conducted a prospective, 1-year single-center study of 103 RA patients in remission to evaluate the predictive performance of calprotectin through a survival analysis and Cox regression models, in order to analyze the effect of covariates, similar to our study. As in our JIA study population, they found that in the multivariate analysis, only baseline calprotectin levels independently predicted RA disease relapse [33].

Additionally, in our study, the higher calprotectin values found in patients with a subsequent flare-up were not skewed by possible systemic forms because we had excluded patients with nsJIA. Serum calprotectin could therefore be a marker for residual disease activity, even in the absence of clinical or biological signs of persistent inflammation. In addition, serum calprotectin is directly released at the site of inflammation after macrophage activation and strongly correlates with local disease processes in joints, even when no systemic involvement occurs. Being a small molecule, calprotectin easily diffuses from synovial fluid and inflamed synovium into the blood stream, where can be easily dosed [11,12,13]. It therefore correlates with “immunological” flogosis, which is not detectable in joint objective examination. Indeed, this biomarker may help to redefine the “target” as an “immunologic and imaging remission” other than a clinical one alone, with possible important prognostic implications [9,10,35]. 

We suggest that calprotectin might play a role in patient follow-up in identifying the subjects under treatment in remission who are likely to remain in prolonged remission. The discontinuation or tapering of the treatment can then be discussed without risk of future relapses. This aspect might have great importance for the “tight control” of current JIA management.

More recently, Hinze et al. [24] found different findings from ours when following patients with a polyarticular course treated with TNF inhibitors. However, this study evaluated the predictive value of calprotectin levels in patients only undergoing anti-TNF therapy, and followed up for a period of 6 months and again at the time of long-term treatment discontinuation [24]. In contrast, our prospective study evaluated serum calprotectin in JIA patients, mostly oligoarticular, both on and off therapy and with a longer follow-up time of 18 months. Therefore, the findings are not comparable. In another recent study, the authors found no relationship of serum calprotectin levels with the prediction of treatment response or flares. In this study, the authors excluded nsJIA patients, but included two cohorts of newly onset, DMARD-naive arthritis, making the study cohort very different compared to ours [19]. 

The third key point of our study is the confirmation of serum calprotectin as a potential marker of good treatment response (for all types of combined treatment), also shown in two cohort studies with patients treated with MTX or TNF inhibitors [16,18,30,31]. In our study, the calprotectin levels at baseline in patients who went into remission were higher than those who remained active during the follow-up. We observed by survival analysis a significant difference in remission outcomes among active patients starting with a cut-off of 10 ng/mL at baseline. Similarly, La et al. [34] found higher calprotectin levels at baseline in treatment responders over a 9-month follow-up period with no difference in outcome between patients treated with MTX and those treated with biologics. 

The reason for a greater therapeutic response to DMARDs in patients with higher calprotectin values, a fact already showed in other studies but is controversial, is not currently explained exhaustively in the literature. We can postulate that, since S100A8/A9 and S100 A12 secretion is greatly stimulated by the TNF released from inflammatory cells, and anti-TNF therapy downregulates S100 protein expression, higher levels of calprotectin cause a “receptive” inflammatory state to such modulating drugs [36]. In addition, proteomic studies analysis showed that calprotectin is overexpressed in peripheral blood mononuclear cells (PBMC) of RA patients responding to MTX/etanercept [37]. Therefore, higher pre-treatment values might predict a future DMARD-drugs response [34,37]. The calprotectin cut-offs which we analyzed and found significant to predict relapse or treatment response are much lower than those previously investigated in the literature. However, before individual or panels of biomarkers should be adopted in clinical practice, further efforts are needed to standardize the ELISA methods used and define biological marker cut-off points [9,21,33,38].

In summary, there is abundant evidence that a constitutive activation of innate immunity underlies the pathogenesis of JIA, as evidenced by overexpression of macrophage-derived cytokines (TNF-α, IL1 and IL6), resulting in secondary selection of self-antigen specific T and B lymphocytes of adaptive immunity. Calprotectin, directly released from macrophages in situ, could identify, on the one hand, a subgroup of patients in which the innate component of immunity is predominant and who have greater probability of responding to DMARDs (MTX and anti-TNF-α), which act on this as the first effect [16]. On the other hand, higher calprotectin levels could be an indicator of underlying subclinical innate immunity activation in JIA patients with clinical remission, thus increasing the risk of disease relapse.

The major strength of this study is that it is a rather large prospective study including JIA patients with longitudinal measurement of outcomes (relapse or remission) and adjustment for relevant confounders. To our knowledge, this is the first prospective study with the longest follow-up. In addition, we defined the outcomes (remission/relapse) based on clinical visits and laboratory tests that were objective measures avoiding information bias which could cause misclassification.

Some methodological limitations should be discussed. Firstly, our study population showed a great heterogeneity for the inclusion of JIA subgroup types, the treatment started at baseline and during follow-up, and the different previous treatment. This might have compromised the homogeneity of our sample and probably influenced our findings. Secondly, the small sample could have affected the power of our study. However, it could be considered acceptable evaluating the reported incidence and prevalence of ns-JIA in European and North American populations (from 2.6 to 23 cases per 100,000 and from 16 to 150 per 100,000, respectively) [39]. Thirdly, as in any longitudinal prospective study, our population was subjected to a lack in follow-up. This lack in follow-up could lead to bias if the associations of serum calprotectin levels with remission/relapse outcomes in JIA patients were different between those included and not included in the study. This could lead to both an under- or over-estimation of the associations, and, although this is unlikely, it cannot be excluded. In particular, active JIA patients did not reach the last time point, making a comparison between the two groups difficult. Moreover, although we have not recruited JIA patients presenting further inflammatory and/or infectious disease at baseline, we must consider, given the ubiquity of neutrophils and macrophages and S100 proteins, a possible confounding effect of an activation outside the joint inflammatory site. Lastly, although we adjusted for several confounders, we might not have had information on all possible confounding factors (residual confounders). Although some of their effects might be minimal and are highly correlated with presently used confounders, they could potentially have a substantial effect given the small prevalence of JIA.

Further larger prospective studies with longer follow-up periods and more characterized JIA samples are needed in the near future to understand which biological markers should be used to better manage these patients.

In conclusion, in our cohort study, serum calprotectin was confirmed as a very useful tool for precise identification of the disease state in polyarticular and oligoarticular JIA subtypes. Additionally, serum calprotectin levels might be of help in defining disease outcomes and can be considered as a potential warning of relapse. Therefore, we would suggest that it might be considered as a good biological marker to detect the residual disease activity, even in the absence of clinical or biological signs of persistent inflammation.

However, in daily clinical practice, the role of serum calprotectin in stratifying JIA disease and guiding therapeutic decisions towards safer and more cost-effective strategies is not yet fully understood.

## 4. Materials and Method

This longitudinal, prospective single-center study included 111 ns-JIA [22] patients (55 active patients and 56 inactive patients) and a control group of 50 healthy subjects. 

The study protocol was approved by the Ethics Board of Chieti-Pescara Health Service (MGB_AIG protocol with ethics committee approval on 13 May 2020) and was in line with the ethical standards laid down in the 1964 Declaration of Helsinki. The consent form was signed by parents or legal guardian. 

Patients and controls were enrolled from January 2020 through to September 2021, from subjects who were referred to the Rheumatology Unit of the Department of Pediatrics, University of Chieti, with 18 months of follow-up. Controls were recruited among those who attended the Pediatric Department for minor diseases (trauma or syncope).

All data were stored in a password protected database.

Two subgroups of JIA patients were included: patients with active disease and patients with inactive disease. The inclusion criterion was the diagnosis of any subtype of JIA according to ILAR classification, except for systemic onset of JIA. The exclusion criteria were a diagnosis of systemic JIA and steroid therapy at enrollment time or in the previous four weeks. The case study population included 91 females (81.9%) and 20 males (18.1%) with a diagnosis of JIA. A total of 56 patients had inactive disease according to Wallace criteria for at least 3 months [40] and 55 had clinically active disease. The inclusion criteria for healthy subject enrollment were the absence of inflammatory/infectious disease either currently or in the previous two weeks and the absence of autoimmune diseases. Of these subjects, we further excluded two subjects who developed celiac disease later and one subject whose blood samples were not stored adequately from the analysis. Informed consent was obtained from all children, parents or guardians as appropriate. 

The flow chart study design is described in Figure 1.

### 4.1. Definitions

The disease was defined as inactive according to Wallace criteria when the following criteria were satisfied: no joints with active arthritis; no fever, rash, serositis, splenomegaly or generalized lymphadenopathy due to JIA; no active uveitis; normal acute phase reactants; and a global assessment by a physician indicating no disease activity [3,40]. To define clinical remission *on* medication, each patient had to manifest an active disease for a period of 6 continuous months. Clinical remission *off* medication was defined when the criteria for inactive disease were met for a minimum of 12 continuous months while not taking all anti-arthritis and anti-uveitis medications [40]. A disease flare was the occurrence of any sign of active arthritis and/or active symptoms, when any criteria for inactive disease were no longer met. Remission after treatment was defined as the achievement of inactive disease according to Wallace criteria [17,40].

### 4.2. Clinical Assessment

In this study, at baseline visit (T0) each patient underwent a clinical assessment by an expert pediatric rheumatologist, accredited by PRINTO. For each patient, twenty-seven joints were assessed for swelling, tenderness/pain on motion and restricted motion according to a standard technique [41]. A joint with active arthritis was defined as having swelling or, if no swelling was present, tenderness/pain on motion and restricted motion. At baseline, the following data were recorded for each patient: gender; weight; height; BMI; age at disease onset; age at enrollment; disease duration; ILAR category; antinuclear antibody and rheumatoid factor status; course of joint disease; history of uveitis; previous intra-articular corticosteroid injections; JIA medications received prior to and at the beginning of the study, with its relatively duration; the time between disease onset and MTX or biologic treatment start (the period between MTX beginning and biological therapy start); and the presence of other concomitant autoimmune diseases. 

In the longitudinal phase of the study, the same clinical evaluation was performed by the same PRINTO pediatric rheumatologist at every timepoint (T1, T2, T3 and T4) (Figure 1).

JIA disease activity was assessed at each time-point (T0,T1,T2,T3 and T4) by the Juvenile Arthritis Disease Activity Score (JADAS), a validated score adopting four criteria: (1) number of active joints; (2) a global assessment of disease activity by a physician measured on a 10 cm visual analog scale (VAS), where 0 means no activity and 10 means maximum activity; (3) parent/patient global assessment of well-being measured on a 10 cm VAS, where 0 means very well and 10 means very poor; and (4) normalized ESR. We used the 27-joint reduced count (JADAS-27), which has been found to be a good surrogate for the whole joint count in JIA, with 0 corresponding to total remission and 57 to maximum disease activity [42,43].

### 4.3. Laboratory Examination

Morning blood samples were collected from patients after 12 h of fasting on the same day of the clinical examinations. Complete blood count, inflammation indices (ESR and CRP), serum ferritin and calprotectin were assayed at baseline (T0) in active/inactive JIA patients and in control subjects. The same laboratory examination was performed during every (T1, T2, T3 and T4) evaluation time point for JIA patients in the longitudinal phase. CRP was measured with a quantitative immunoturbidimetric assay with normal values in the range of 0.00–10.00 mg/L. ESR was calculated using the Westergren technique. The normal range values were 0–15 mm/h in males and 0–20 mm/h in females. Ferritin was measured with a quantitative chemiluminescence immunoassay, with normal range values between 4.6 and 204 ng/mL. Albumin was measured with a colorimetric assay. Serum and plasma samples were immediately centrifuged and within two hours all sera were stored at −20 °C until analysis. Serum calprotectin of each patient was detected by Calprest^®^NG, an immunoenzymatic assay, from Eurospital diagnostics. The kit sensitivity, specificity and negative predictive value declared by the manufacturer are 94.6%, 99.2% and 89.8, respectively, and the range of the normal value was 0–3 ng/mL.

### 4.4. Statistical Analysis

Continuous data were expressed as means ± standard deviation (SD) or median (5–95% range), and categorical data were presented as percentage and count. Anthropometric data, such as body mass index (BMI), was expressed as z score, calculated by using the SIEDP (Italian Society for Pediatric Endocrinology and Diabetology) growth calculator 3.0 online version, which is based on the work of Cacciari et al. [44].

We compared the characteristics of inactive and active JIA patients by using independent sample T-tests, Mann–Whitney U and Pearson’s Chi-square tests. We used one-way ANOVA and Kruskall-Wallis tests to compare active and inactive JIA patients and controls. The serum calprotectin biomarker was dichotomized using a threshold level of 3 ng/dL, which represents the upper limit of normality in healthy controls in our laboratory. Consecutively, we applied progressive cut-off levels, starting from 3 ng/dL, to investigate the effect of different values of serum calprotectin on the outcome (flare/remission) during the follow-up of the patients. The time from enrollment to disease relapse or remission in inactive and active JIA patients was evaluated by survival analysis in a follow-up period of maximum 18 months. In the inactive JIA group, patients without relapse within 18 months were censored and considered to be in stable remission. In the active JIA group, patients without remission within 18 months were censored and considered to be patients in active disease unresponsive to current treatment. Furthermore, Kaplan–Meier analysis was performed to estimate relapse or remission-free survival in patients with lower versus (vs.) higher serum calprotectin levels. In every group of JIA patients (inactive/active), associations of clinical factors at baseline (serum calprotectin (ng/dL), gender, BMI z score, age at onset of disease, duration of disease (years), MTX duration (years), biological drugs duration (years) and therapy at baseline) with disease relapse or remission were assessed using Cox proportional hazard regression models. Crude and adjusted hazard ratios (HRs) with 95% confidence intervals (CIs) were estimated. Similarly, multivariate models were constructed to analyze the effect of covariates and to fully adjust the association of serum calprotectin with relapse or remission of disease. First, confounders were selected from the literature and were subsequently tested for their association with both the determinant and the outcome or a change in the unadjusted effect estimates of 10% when added to the univariate model [35,36,45,46,47,48]. Confounders were included in the final model if they were either associated with determinant and outcome, not in the causal pathway, or if the effect estimate changed by 10% when they were included.

Point estimates were provided with 95% CI. The statistical significance level was *p* < 0.05. SPSS version 25.0 for Windows (IBM, Armonk, NY, USA) and STATA/IC 15.1 (Stata Corp. 2017. *Stata Statistical Software: Release 15*. StataCorp LLC., College Station, TX, USA) were used to perform statistical analyses.

## Figures and Tables

**Table 1:** Demographic, clinical and laboratory data of the study population.

	**Inactive JIA** **(*n* = 56)**	**Active JIA** **(*n* = 55)**	**Controls** **(*n* = 47)**	**# *p* Value**
**Sex, Female (%)**	48 (85.9)	43 (78.0)	31 (66.0)	0.090
**Age at enrollment** **(ys)**	10.5 (3.20–17.18)	11.9 (5.53–16.30)	8.9 (2.27–15.3)	**§ 0.011**
**BMI (Kg/m^2^)**	18.7 (14.00–28.50)	19.1 (14.00–26.00)	18.4 (13.00–24.00)	**§** 0.345
**BMI z-score**	0.0 (1.06)	0.4 (0.23)	0.4 (0.77)	* 0.130
**Previous uveitis (%)**	43 (76.8)	44 (88.0)	N/A	0.576
**JIA subgroup (%)** *Oligoarthritis* *Oligoarthritis-extended* *Polyarthritis* *Psoriasic arthritis* *Entesitis-arthritis*	31 (55.4) 11 (19.6) 11 (19.6) 3 (5.3) 0 (0.0)	27 (54.0) 8 (14.0) 11 (20.0) 5 (10.1) 1 (2.0)	N/A	0.486
**Ongoing therapy at baseline (%)** *MTX* *Etanercept* *Adalimumab* *MTX + adalimumab* *MTX + etanercept* *Infliximab* *NSAIDs* *No therapy*	29 (51.8) 4 (7.1) 6 (10.7) 7 (12.5) 1 (1.8) 1 (1.8) 0(0) 8 (14.3)	28 (50.9) 2 (3.6) 2 (3.6) 0 (0) 0 (0) 0 (0) 6 (10.9) 12 (21.8)	N/A	N/A
**New therapy at baseline (%)** *MTX* *Adalimumab* *Etanercept* *Infliximab*	N/A	20 (36.3) 15 (27.3) 11 (20) 4 (7.3)	N/A	N/A
**N. of active joints at** **the onset of JIA**	3.1 (2.93–16.00)	2.2 (1.76–12.00)	N/A	**§** 0.092
**Disease duration (ys)**	5.0 (0.00–12.00)	4.9 (0.00–9.81)	N/A	**§** 0.387
**JADAS-27**	0.0 (0.00–7.00)	5.9 (2.50–18.00)	N/A	**§ <0.001**
**Positive ANA (%)**	11 (24.4)	15 (71.3)	N/A	0.210
**TMj** **Inflammation (%)**	0 (0.0)	13 (26.5)	N/A	**<0.001**
**Laboratory Data**				
**Laboratory value** **(normal range)**	**Inactive JIA** ***n* = 56**	**Active JIA** ***n* = 55**	**Controls** ***n* = 47**	**§ *p* value**
**Hb (g/dL)** **(12–16)**	13.25 (12.78–16.00)	12.80 (10.60–15.00)	13.40 (11.60–16.70)	0.968
**PLT/µL** **(150,000–450,000)**	290,500 (188,000–410,000)	325,000 (202,000–437,000)	245,282 (202,000–437,000)	0.053
**Leucocytes/µL** **(4000–10,000)**	6,845 (1240–5870)	6,495 (4220–9400)	5910 (1319–6210)	0.360
**Neutrophils/µL** **(2000–7000)**	3054 (1,228–6980)	3084 (1150–5420)	3150 (1150–5380)	0.764
**Lymphocytes/µL** **(1000–3500)**	2675 (1620–5870)	2575 (1913–5320)	2100 (319–6210)	0.167
**Ferritin (ng/mL)** **(4.6–204)**	29.61 (18.70–64.80)	26.50 (8.70–69.80)	37.09 (12.30–65.74)	0.069
**Calprotectin** **(ng/mL)**	3.30 (0.60–38.8)	7.00 (1.20–34.00)	4.00 (1.70–7.20)	**0.018**
**Albumin (g/dL)** **(3.5–5.5)**	4.42 ± 0.21	4.39 ± 0.23	4.44 ± 0.17	0.230
**ESR (mm/h)** **(2–15)**	7.00 (3.00–9.00)	7.50 (3.00–23.00)	7.60 (2.00–10.00)	0.050
**CRP (mg/dL)** **(0.00–10.00)**	0.29 (0.17–6.60)	0.45 (0.10–9.50)	1.35 (0.29–5.10)	0.051

Data are expressed as means (SD), median and range (5–95%) or absolute numbers and percentages (%). N/A: not applicable; N: number; JIA: juvenile idiopathic arthritis; yr: years; JADAS-27: Juvenile Arthritis Disease Activity Score; RF: rheumatoid factor; HLA-B27: human leukocyte antigen B27; TMj: temporomandibular joint.; ESR: erythrocyte sedimentation rate; CRP: C reactive protein; BMI: body mass index; MTX: metothrexate; NSAIDs: non steroid anti-inflammatory drugs. Bold indicates values where the *p*-value is <0.05. # *p* value from Chi-squared test; § *p* value from Kruskal–Wallis test; * *p* value from one-way ANOVA.

**Table 2:** Associations of different cut-off levels of serum calprotectin with the risk of relapse or remission in inactive and active JIA patients, respectively.

Inactive JIA Patients (Relapse vs. Non-Relapse)	Relapse HR (95% CI)	Active JIA Patients (Remission vs. Non-Remission)	Remission HR (95% CI)
(cal. ≥ 3 vs. cal. < 3)		(cal. ≥ 3 vs. cal. < 3)	
CRUDE MODEL *p*-value	1.43 (0.86, 2.42)) 0.171	CRUDE MODEL *p*-value	0.75 (0.47, 1.21) 0.242
* CONFOUNDER MODEL *p*-value	2.19 (1.20, 4.10) **0.011**	CONFOUNDER MODEL *p*-value	0.74 (0.43, 1.27) 0.271
(cal. ≥ 10 vs. cal. < 10)		(cal. ≥ 10 vs. cal. < 10)	
CRUDE MODEL *p*-value	1.54 (0.92, 2.59) 0.104	CRUDE MODEL *p*-value	0.44 (0.26, 0.75) **0.002**
CONFOUNDER MODEL *p*-value	1.86 (1.06, 3.26) **0.030**	CONFOUNDER MODEL *p*-value	0.46 (0.26, 0.80) **0.006**
(cal. ≥ 12 vs. cal. < 12)		(cal. ≥ 12 vs. cal. < 12)	
CRUDE MODEL *p*-value	1.66 (0.96, 2.84) 0.068	CRUDE MODEL *p*-value	0.44 (0.26, 0.77) **0.004**
CONFOUNDER MODEL *p*-value	1.99 (1.10, 3.62) **0.023**	CONFOUNDER MODEL *p*-value	0.47(0.26, 0.84) **0.011**
(cal. ≥ 15 vs. cal. < 15)		(cal. ≥ 15 vs. cal. < 15)	
CRUDE MODEL *p*-value	1.75 (1.01, 3.03) **0.046**	CRUDE MODEL *p*-value	0.44 (0.23, 0.86) **0.016**
CONFOUNDER MODEL *p*-value	1.94 (1.08, 3.48) **0.028**	CONFOUNDER MODEL *p*-value	0.45 (0.22, 0.94) **0.033**
(cal. ≥ 20 vs. cal. < 20)		(cal. ≥ 20 vs. cal. < 20)	
CRUDE MODEL *p*-value	2.01 (1.11, 3.66) **0.021**	CRUDE MODEL *p*-value	0.50 (0.26, 0.98) **0.042**
CONFOUNDER MODEL *p*-value	2.07 (1.07, 4.00) **0.031**	CONFOUNDER MODEL *p*-value	0.54 (0.26, 1.29) 0.101

Data are presented as change in HR derived from the Cox regression model; the reference category is represented by JIA patients with serum calprotectin <3, <10, <12, <15 or <20 for different cut-off levels for both groups; calprotectin is expressed in ng/mL. cal.: calprotectin. * additionally adjusted for sex, age at onset of the disease, body mass index (BMI) sds, disease duration, methotrexate treatment duration, biologic drug treatment duration and therapy at baseline. Bold *p* value < 0.05; HR: hazard ratio.

## Data Availability

Not applicable.
